# Migration of 35 Siloxanes from Silicone Food Contact Materials in China and Their Potential Exposure Assessment

**DOI:** 10.3390/foods15081387

**Published:** 2026-04-16

**Authors:** Ya Chen, Hongyan Li, Haizhi Huang, Xuping Shentu

**Affiliations:** 1 Key Laboratory of Consumer Goods Safety Research, Zhejiang Provincial Bureau of Market Supervision and Administration, Zhejiang Institute of Quality Sciences, Hangzhou 310018, China; yachen_alisa@yeah.net; 2 Key Laboratory of Microbiological Metrology, Measurement & Bio-Product Quality Safety, State Administration for Market Regulation, China Jiliang University, Hangzhou 310018, China; huangtian205@163.com (H.H.); stxp@cjlu.edu.cn (X.S.)

**Keywords:** cyclic methyl siloxanes, linear methyl siloxanes, food contact materials, age-stratified exposure assessment, risk quotient, threshold of toxicological concern

## Abstract

Silicone food contact materials (FCMs) pose potential health risks due to the migration of siloxanes. This study presents a comprehensive migration profiling of 35 siloxanes (cyclic D3–D22 and linear L2–L14) from 30 silicone FCMs, with migration tests rigorously conducted under worst-case intended-use scenarios to ensure conservative and reliable exposure estimates. Methodological innovations include an expanded analytical scope, age-stratified exposure assessment across seven age groups, and a multi-tiered risk evaluation framework. The results reveal that migration behaviors were affected by simulant polarity, siloxane solubility, and silicone thermal stability. The risk evaluation framework integrates aggregate migration limits for total cyclic (D3–D13) and total cyclic plus linear siloxanes (D3–D13, L3–L13), complemented by individual siloxane assessment via Risk Quotient (RQ) and Threshold of Toxicological Concern (TTC) approaches. While the total migration of cyclic siloxanes exceeded the proposed action limit of 12 mg/kg for adults in several samples and 2 mg/kg for children in most samples, granular assessment revealed divergent risks: Cyclic D4 and D5 showed negligible risk (RQ < 5). In contrast, D3 migration posed a potential concern (RQ > 5), especially for individuals aged >13 years. Notably, the estimated exposures to 14 siloxanes with low molecular weight (<1000 Da), including highly prevalent D6 and L12 with detection frequency >90%, exceeded the TTC threshold across all age groups, highlighting unaddressed risks that are not captured by aggregate action limits. This work underscores the need for substance-specific, age-specific risk evaluations and regulatory updates for silicone FCMs.

## 1. Introduction

Siloxanes, characterized by a repeating Si-O-Si backbone, are typically categorized into three groups: linear methylsiloxanes (LMSs), cyclic methylsiloxanes (CMSs), and various derivatives (e.g., halosiloxanes and organofunctional siloxanes). A substantial body of evidence demonstrates that certain siloxanes bioaccumulate in biological systems and exert adverse effects, including disruption of the nervous, immune, and endocrine systems, potentially leading to connective tissue disorders and organ dysfunction [1,2,3]. Consequently, worldwide research attention has increased, and global regulatory scrutiny has been intensified [4,5]. For instance, the European Union (EU) has classified D4 as both a Persistent, Bioaccumulative, and Toxic (PBT) and a very Persistent and very Bioaccumulative (vPvB) substance, while D5 is classified as a vPvB substance [6,7,8]. Furthermore, D4, D5, and D6 are listed as Substances of Very High Concern (SVHCs) under the EU’s Registration, Evaluation, Authorization and Restriction of Chemicals (REACH) regulation and are under evaluation for inclusion in the annex(es) of the Stockholm Convention on Persistent Organic Pollutants (POPs) [9] (see Table 1 for details).

Silicone is a widely used material for FCMs due to its favorable surface properties, physiological inertness, thermal stability, and antioxidant capacity [10,11,12]. However, the migration of components, particularly siloxanes, into food poses potential health risks [13,14,15,16,17,18]. Although pre-market safety evaluations in many countries cover starting substances and additives and cyclic siloxanes such as D4 and D6 are subject to regulation, significant knowledge gaps remain. LMSs (L2–L14) and CMSs (D7–D22) remain understudied, lacking comprehensive toxicological data and systematic exposure assessments despite their inherent hazard potential [19,20]. This is particularly concerning for infants and young children, a primary consumer group for silicone FCMs, whose developing physiological systems heighten their vulnerability to chemical exposures [21,22,23]. Moreover, factors such as repeated use, material aging, and the potential presence of degradation byproducts can enhance the migration of siloxanes from silicone FCMs. Consequently, both children and adults who routinely use silicone-based FCMs face sustained potential dietary exposure risks.

The migration of siloxanes from silicone FCMs is a complex process influenced by material properties, manufacturing processes, and food type [24,25,26,27,28,29]. Existing research has primarily focused on children’s silicone products like pacifiers, teething rings, and soft rubber toys that involve oral contact. Although the recent landmark study by Wrona et al. [30] conducted a thorough safety assessment of 44 silicone baking molds from multiple European countries (supplied by the International Consumer Research and Testing Limited), providing critical insights into the migration patterns of siloxanes and other volatile compounds from EU-market silicone products during high-temperature baking, significant research gaps remain. Specifically, there is a lack of systematic investigations into the migration characteristics and full-age-stratified exposure assessment of the wide range of silicone FCMs available through mainstream sales channels in China, alongside a notable shortage of quantitative studies characterizing siloxane exposure via these FCMs. Lin Xu et al. [4] investigated methylsiloxanes in silicone children’s products sold in China, detecting 14 methylsiloxanes (including D4, D5, and L5) in saliva extracts. Di Feng et al. [5] conducted identification, migration studies, and childhood exposure assessments of methylsiloxanes in silicone baby pacifiers and found that children’s daily oral intake of methylsiloxanes (MW < 1000) ranged from 52 to 146 μg/kg bw/day. Oral ingestion, particularly via infant formula, may be the primary route of methylsiloxane exposure for children. In contrast, critical gaps remain in understanding siloxane migration from different types of silicone FCMs across diverse usage scenarios and in quantifying the associated exposure risks for different consumer groups.

Existing research has confirmed that siloxane residues are commonly found in silicone FCMs; however, there are limited systematic studies on migration patterns and exposure assessments across all age groups for relevant products sold in China (particularly those intended for infants and young children). Furthermore, in China, silicone FCMs distributed through e-commerce platforms account for a major segment of FCM sales; therefore, conducting research on products sold through this channel holds direct public health significance. To address the aforementioned research gaps, this study examined 30 silicone FCMs using a worst-case experimental design, with the overarching aims of: (1) qualitatively and quantitatively characterizing siloxane profiles in the test materials; (2) analyzing their migration behaviors under aggressive simulated usage conditions; and (3) conducting a multi-dimensional exposure risk assessment across distinct consumer groups. This rigorous simulation framework was designed to establish a conservative upper bound for safety assessment, providing a baseline reference for the development of regulatory thresholds rather than fully replicating routine daily use scenarios for consumers. Innovatively, the target population was stratified into seven age subgroups based on physiological development, enabling age-specific exposure assessment using age-specific parameters from the U.S. Environmental Protection Agency (EPA) Exposure Factors Handbook [31]. Methodologically, the risk assessment was tailored to meet diverse data and regulatory requirements, including evaluations of total migration of cyclic siloxanes (D3–D13) and total cyclic plus linear siloxanes (D3–D13 and L3–L13) against proposed action limits as well as exposure assessment of individual siloxanes via the RQ and TTC methods.

## 2. Materials and Methods

### 2.1. Chemicals

A total of 17 siloxanes (L2–L8, D3–D9, L10, MDHM, INCI; specific standard information is provided in Table 1) were dissolved in chromatographic-grade methanol or n-hexane, 34 n-alkane standard mixture (C7–C40, 1000 μg/mL in n-hexane), and n-eicosane (C20), and these were purchased from Anpu Cuishi Standard Technology Service Co., Ltd. (Shanghai, China) and Tianjin Alta Technology Co., Ltd. (Tianjin, China), respectively. Chromatographic-grade n-hexane, methanol, and dichloromethane were purchased from Thermo Fisher Scientific Co., Ltd. (Shanghai, China). Analytical-grade ethanol, anhydrous sodium sulfate, sodium chloride, and acetic acid were supplied by China National Pharmaceutical Group Chemical Reagent Co., Ltd. (Beijing, China). The working solutions were prepared in methanol and n-hexane and stored in the dark at −18 °C.

### 2.2. Sample Preparation

Representative sampling of silicone FCMs was performed to reflect the primary distribution channels of these products in China. A total of 30 silicone FCM samples were collected via stratified random sampling from major Chinese e-commerce platforms, including Taobao, JD.com, and Pinduoduo. The sample set included products from five top-selling domestic brands, seven imported brands, and ten regional manufacturers, ensuring broad market representativeness. As illustrated in Figure S1 (Supplementary Materials), the collected samples covered common categories such as molds, bowls, spoons, and straws and originated from multiple geographical regions. This selection aimed to cover the predominant products available online, thereby enhancing the representativeness of the silicone FCMs.

Prior to migration experiments, each silicone sample was prepared under controlled conditions. Representative sections of the material intended for direct food contact were cut using stainless steel scissors. To ensure consistency and comply with surface-area-to-volume ratio requirements, samples were trimmed into uniform strips measuring 6 cm^2^ and stored in sealed inert containers before analysis.

### 2.3. Dissolution Profiling of Siloxanes

Measured (1 ± 0.001) g samples of the silicone FCMs were added to glass centrifuge tubes. Each sample was extracted with 10 mL of acetone as the extraction solvent. The mixtures were then subjected to ultrasonic extraction for 15 min to facilitate the release of siloxanes, after which the extracts were filtered through 0.22 μm PTFE filters. All extractions were performed in triplicate, with procedural blanks processed in parallel under identical conditions.

### 2.4. Migration Experiment

The migration tests in this study were conducted in accordance with the EU General Framework Regulation on FCMs (Regulation (EU) No. 1987/2019) [32] and the EU harmonized test conditions for kitchenware FCMs [33] and were designed and carried out in accordance with relevant Chinese national standards, including GB 4806.1-2016, GB 31604.1-2023, GB 4806.11-2016, and GB 31604.8-2021 [34,35,36,37] while employing established methods for the analysis of siloxanes [38].

Based on a systematic evaluation of all intended-use scenarios for the tested FCMs—including potential food types, contact temperatures, and contact durations—the most severe migration conditions were selected for testing and safety assessment (detailed migration conditions are provided in Table S1). This worst-case exposure approach, a globally accepted protocol for FCM compliance assessment, was applied to cover the most stringent contact scenarios throughout the entire product life cycle (e.g., high-temperature frying, long-term contact with high-fat foods), thereby ensuring the conservatism and reliability of the safety assessment. For each material, food simulants were selected to represent the most stringent real-world exposure for each food category: 4% (*v*/*v*) acetic acid for aqueous foods, 50% (*v*/*v*) ethanol for alcoholic foods, and 95% (*v*/*v*) ethanol for fatty foods. All migration tests were performed at 70 °C for 2 h, defined as the worst-case exposure condition in this study.

Each 6 cm^2^ sample strip was fully immersed in a glass vessel containing 10 mL of the corresponding simulant. After migration, 1 mL aliquots of the 95% (*v*/*v*) ethanol simulant were pipetted and mixed with 1 g of anhydrous sodium sulfate for complete dehydration. For the 4% (*v*/*v*) acetic acid and 50% (*v*/*v*) ethanol simulants, 2 mL aliquots of each simulant were transferred to separate vessels and extracted three times with 2 mL of dichloromethane. After phase separation, the lower organic layers were collected, transferred to volumetric flasks, and diluted to a final volume of 2 mL for subsequent analysis. All extracts were then filtered through 0.22 μm PTFE filters. All tests were performed in triplicate.

### 2.5. Gas Chromatography–Mass Spectrometry (GC-MS) Analysis

Quantitative analysis was performed using an Agilent 7890B gas chromatograph coupled to a 7000C triple quadrupole mass spectrometer (Gas Chromatography–Mass Spectrometry, GC-MS) (Agilent Technologies, Santa Clara, CA, USA). Separation was achieved on a HP-5MS capillary column (30 m × 0.25 mm i.d. × 0.25 μm film thickness). Helium was used as the carrier gas at a constant flow rate of 1.0 mL/min. Samples were injected in splitless mode at 280 °C. The oven temperature program was as follows: initial hold at 40 °C for 1 min, ramp to 315 °C at a rate of 10 °C/min, and final hold at 315 °C for 15 min. The mass spectrometer was operated in Electron Ionization (EI) mode at 70 eV. Data were acquired in full-scan mode (*m*/*z* 50–1000) and selected ion monitoring (SIM) mode for qualitative and quantitative analysis, respectively. Molecular weights (MWs) and characteristic monitoring ions for all targeted siloxanes are listed in Table 1.

### 2.6. Qualitative and Quantitative Analysis

Qualitative identification of the 35 siloxanes was performed by combining retention time (RT) matching, mass-spectral characteristics, and retention index (RI) comparison. A mixed standard solution containing 17 commercially available siloxanes (L2–L8, D3–D9, L10, MDHM, and INCI) was prepared in n-hexane at gradient concentrations. Analytes were identified by comparing their RTs and mass spectra with those of the reference standards and quantified using external calibration curves. For the remaining 18 siloxanes (L9, L11–L14, D10–D22), RI values were calculated using Formula (1) and validated against the literature or database records. A surrogate calibration approach was established using L5 and D6 as representative standards for linear and cyclic siloxanes, respectively, as described in the literature [39].

(1) RI = 100z + 100 × [(t_R(x)_ − t_R(z)_)/(t_R(z+1)_ − t_R(z)_)]

where t_R_(x) is the RT of the target siloxanes, and t_R_(z) and t_R_(z + 1) are the RTs of n-alkanes with adjacent carbon numbers of _z_ and _z+1_, respectively. To accurately calculate the RIs of siloxanes, a homologous series of n-alkanes (C_7_ to C_40_) was analyzed under the same chromatographic conditions, with C_20_ as the reference to establish a reliable RI calibration baseline.

### 2.7. Method Evaluation and Quality Control

Method performance was evaluated in terms of linearity, sensitivity, and precision. The limits of detection (LOD) and quantification (LOQ) were defined as the concentrations corresponding to signal-to-noise ratios of 3 and 10, respectively. Recovery experiments were conducted by spiking blank food simulants (4% acetic acid, 10%, 50%, and 95% ethanol) at three concentration levels (LOQ, 2 × LOQ, and 10 × LOQ), with each level analyzed in triplicate. All data were processed using Microsoft Excel and OriginPro 2022.

Quality control measures comprised regular calibration and maintenance of instrumentation, analysis of solvent and procedural blanks every 10 injections, and strict adherence to standardized protocols to minimize contamination and operational variability.

### 2.8. Potential Exposure Assessment

Potential exposure to low-molecular-weight siloxanes (MW < 1000) was conducted by integrating migration data with consumption patterns of silicone FCMs.

Exposure to sum of CMSs (D3–D13) and sum of CMSs (D3–D13) and LMSs (L3–L13) was evaluated against the action limits proposed by DTU Food [39]. Furthermore, for risk assessment of exposure to individual siloxane, daily exposure (mg/kg body weight (bw) per day, mg/kg BW/day) was calculated according to Equation (2), according to the approach described by Hu et al. [40].

(2) Exposure = Migration_1_ × A × CF

where migration_1_ (mg/dm^2^) denotes the migration amount per area (dm^2^) under extreme conditions. A (dm^2^/person/day) is the daily contact area derived from the assumption of 1 kg of food intake per person with a contact area of 6 dm^2^, and CF is the consumption factor reflecting the fraction of food packaged in the material of interest. Migration_1_ was converted using Equation (3).

(3) Migration_1_ (mg/dm^2^) = migration_measured_ (mg/kg)/6 (dm^2^/kg)

The CF values were adopted from Liu et al. [41] and U.S. Food and Drug Administration (FDA) guidelines [42]: 0.542 for infants and toddlers aged 0–36 months and 0.4 for children over 36 months. Age-stratified daily intake values were obtained from the EPA Exposure Factors Handbook.

(4) $$\mathrm{RQ}=\frac{Exposure}{RfD}$$

Risk was executed by employing the RQ and the TTC methods. For substances with established reference doses (RfDs) from authoritative toxicological databases (e.g., EPA, ECHA, and PubChem), RQ was computed using the RfDs and exposure values (see Equation (4)). An RQ < 1 indicates no significant risk, 1 ≤ RQ < 5 suggests process optimization may be needed, and RQ ≥ 5 signifies a potential risk. For siloxanes as non-intentionally added substances lacking RfDs, the limit of 0.0015 mg/kg bw/day for Cramer III substances was employed according to the TTC concept [43]. Exposure levels exceeding this threshold were considered to represent a potential concern.

The target population was divided into seven age groups (≤1, 1–3, 3–6, 6–13, 13–20, 20–50, >50 years) to reflect different usage patterns and physiological susceptibilities. Infants and young children (0–6 years) are primary users of products such as silicone pacifiers, bottles, and infant utensils, whereas older groups are exposed mainly through silicone molds, tableware, and straws.

### 2.9. Statistical Analysis

#### 2.9.1. Data Preprocessing

For the quantitative dataset of 35 target siloxanes from solvent extraction and migration tests, unified preprocessing rules were established prior to subsequent analysis. Missing values (no valid chromatographic response) and values below the limit of detection (<LOD) of the validated method were uniformly replaced with 0. This filling strategy was adopted to ensure the integrity of the dataset matrix for multivariate analysis while avoiding the introduction of hypothetical non-detected values that may interfere with the characterization of the actual distribution pattern of siloxanes in tested samples.

#### 2.9.2. Principal Component Analysis (PCA)

Principal component analysis was performed to visualize and verify the product-category-specific siloxane residue patterns in silicone FCMs. No normalization or scaling transformation was applied to the dataset prior to PCA, which was performed directly using the raw quantitative concentration data of target siloxanes. This processing strategy was selected based on the core objective of PCA in this study: to reveal the overall differences in siloxane residue levels and component profiles among different product categories and to identify the dominant characteristic components driving inter-category differentiation. The original concentration data can truly reflect the actual difference in content of various siloxane homologues in the tested samples and highlight the contribution of high-content characteristic components to sample classification, which is fully consistent with the research purpose of this study. The score plot of PCA was used to present the clustering characteristics of different sample categories, with 95% confidence ellipses to show the grouping differentiation.

#### 2.9.3. Migration Behavior Difference Analysis

To characterize siloxane migration behaviors across different food simulants and product categories, descriptive statistical analysis was used to report the detection frequency, migration level range, and overall distribution profile of the target siloxanes.

## 3. Results

### 3.1. Identification of Siloxanes and Method Evaluation

**Figure 1:**
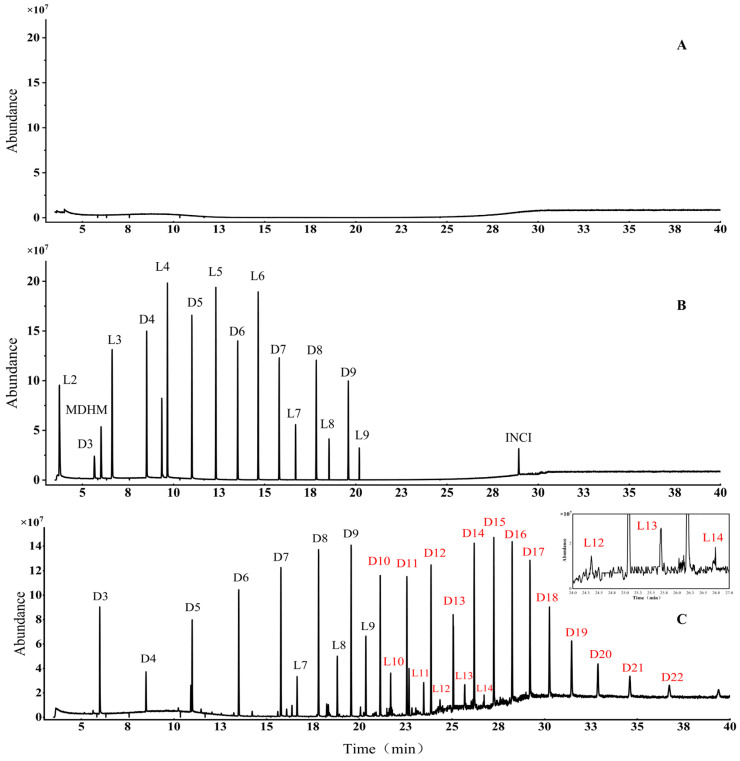
Total ion chromatograms. (**A**) Blank; (**B**) 17 siloxane mixed standards; (**C**) siloxanes detected in a silicone sample. Siloxanes identified by the RI method are marked in red.

Figure 1 shows the total ion chromatograms (TICs) of the blank, the 17 siloxane mixed standards and a representative experimental sample acquired under the same optimized conditions. Well-resolved, alternating chromatographic peaks were observed for LMSs and CMSs, with each homologous series showing a consistent mass increment of 74 Da, corresponding to the (CH_3_)_2_OSi repeating unit, confirming their structural classification. Red-highlighted regions in Figure 1C represent chemicals without corresponding commercial standards. Based on characteristic fragment ions and literature-based structural insights, these are tentatively assigned to uncharacterized LMS or CMS homologues. As shown in Table 1, experimentally determined RIs (RI-Exp.) showed deviations from values in the literature (RI-Doc.) that did not exceed 15, thus confirming a reliable match for identification [5].

The liquid–liquid extraction results indicated that the recoveries of n-hexane, ethyl acetate, a 1:1 (*v*/*v*) mixture of ethyl acetate and n-hexane, a 1:1 (*v*/*v*) mixture of ethyl acetate and cyclohexane, and dichloromethane were 54.81–94.46%, 61.47–92.02%, 59.13–91.83%, 61.54–91.33%, and 69.82–110.12%, respectively. Dichloromethane was selected, and different extraction times (10 s–10 min) were then evaluated. Recovery with the highest efficiencies (75.95–104.58%) was achieved at 1 min. The 17 target siloxanes demonstrated good linear correlation (R^2^ > 0.9913), sensitivity (LOD ≤ 1.0 mg/kg) and stability (Relative Standard Deviation, RSD < 5.9%). Spiked recovery experiments showed that recoveries in different food simulants ranged from 68.05% to 106.10%, with an RSD of 1.5% to 4.3%.

### 3.2. Extraction Profile

The analysis of 30 silicone FCMs revealed the ubiquitous presence of siloxane residues extracted by acetone (see in Table S2), with total concentrations ranging from 174 to 25,046 mg/kg. CMSs (D3-D21) constitute the dominant composition across nearly all samples, while LMSs (L9, L10, L12-L14) were detected less frequently and at notably lower levels, except in a few specific samples (e.g., L10 in S22: 1075 mg/kg, L13 in S18: 5717 mg/kg).

Marked differences in residue profiles were observed across product categories. Silicone molds exhibited the highest overall CMS residue levels (8323–25,046 mg/kg) and a marked predominance of higher-molecular-weight cyclic siloxanes (D10–D16). Bowls/plates and spoons showed high-to-moderate total residues (sum of CMS: 6182–22,268 mg/kg), with a broader distribution of cyclic siloxanes from D4 to D16. Notably, sample 18 (rubber gloves) presented an anomalous profile with an exceptionally high concentration of L13, underscoring category-specific formulation or degradation. These findings align with reports in the literature confirming the universal prevalence of CMSs and substantial inter-product variability [44].

**Figure 2:**
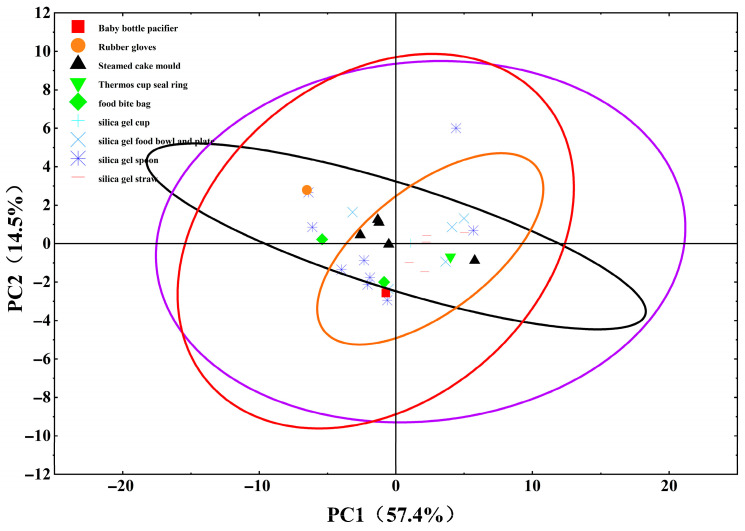
PCA score plot of siloxane residues in solvent extracts of silicone FCMs. The orange circles indicate samples from the “Steamed cake mold” category, the red circles indicate samples from the “Silica gel food bowl and plate” category, the purple circles indicate samples from the “Silica gel spoon” category, and the black circles indicate samples from the “Silica gel straw” category.

To further visualize and verify these product-specific residue patterns, PCA was performed on the preprocessed solvent extraction dataset (detailed data processing rules are provided in Section 2.9), with the resulting score plot shown in Figure 2. PC1 and PC2 accounted for 57.4% and 14.5% of the total variance, respectively, yielding a cumulative explained variance of 71.9%—a level sufficient to capture the core variability in siloxane residue profiles. Consistent with the quantitative results, silicone mold samples (denoted by ▲ in Figure 2) clustered tightly within the corresponding 95% confidence ellipse, reflecting the highly consistent residue characteristics (notably the predominance of higher-molecular-weight CMSs) across this product category. By contrast, bowl/plate and spoon samples (marked by × and ⋆) aggregated within a distinct cluster, which aligns with their broader distribution of cyclic siloxanes (D4–D16) observed in the quantitative analysis. Sample S18 (rubber gloves, ●) deviated distinctly from the main clusters, occupying a separate 95% confidence ellipse; this isolation mirrors its anomalous residue profile (dominated by a high concentration of L13) noted earlier, further underscoring the category-specific formulation or degradation processes. This PCA visualization thus reinforces the conclusion that product type is a key determinant of siloxane residue profiles in silicone FCMs, providing a statistical complement to the quantitative solvent extraction findings.

### 3.3. Migration Profile

**Figure 3:**
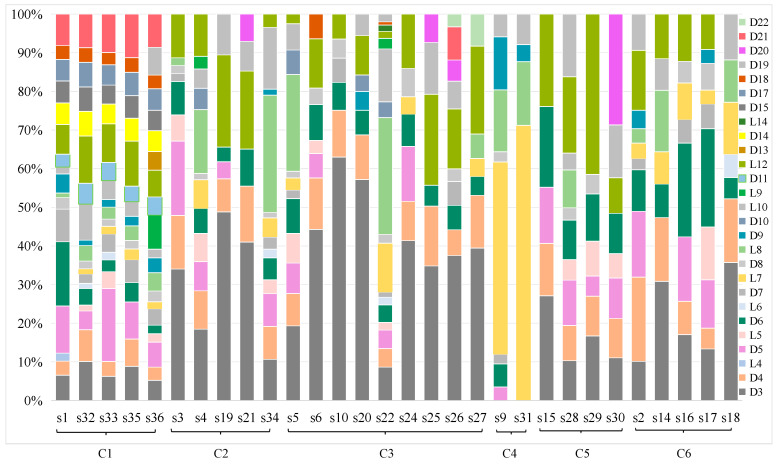
Migration of 27 siloxane compounds under extreme conditions in various FCMs (mg/kg food).

Figure 3 demonstrates that total of 27 siloxanes migrated from the 30 silicone FCMs evaluated into food simulants selected to represent the most severe foreseeable conditions during contact with aqueous, alcoholic, and fatty foods (see details in Table S3).

Analysis of specific migration results revealed that low-molecular-weight cyclic siloxanes (D3–D6) were the predominant migrants, with detection frequencies (DFs, DF = number of positive samples/total samples × 100%) of 93.3%, 93.3%, and 96.7% for D3, D4, and D6 across the 30 samples, respectively. In contrast, siloxanes such as L4, L14, and D22 exhibited very low DFs (≤6.7%), suggesting limited release. Migration profiles were strongly dependent on the food simulant. Marked differences were observed across simulants: multiple siloxanes were detected in most samples exposed to 50% and 95% ethanol, whereas negligible migration was observed in 4% acetic acid. This simulant-dependent migration pattern is in good agreement with previous reports, where alcoholic food simulants showed significantly higher solubilization capacity for both cyclic and linear siloxanes from silicone matrices, while acidic aqueous matrices induced negligible siloxane release [30]. The simulant with 95% ethanol was the most aggressive, yielding the highest migration levels for cyclic siloxanes across all tested samples (e.g., D6 reached 7.51 mg/kg in S1, and D5 reached 7.11 mg/kg in S33). Variability among sample types was also evident; for instance, the silicone spoon of S22 exhibited notably higher migration of several siloxanes (e.g., L8, L10, and D10) in 50% ethanol compared to others. It is noteworthy that migration into the 4% acetic acid involved higher linear siloxanes of L7 and L8.

Compared to the solvent extraction profiles, siloxane migration levels into the food simulants were substantially lower, indicating that only a small fraction of the total siloxane content was released. Notably, several LMSs (e.g., L4, L5, L7, L8) were detected in simulants but were absent in acetone extracts, likely via silicone degradation under high temperature. This suggests that migration is highly selective and influenced by simulant properties, siloxane solubility, and silicone thermal stability. These findings address key scientific questions regarding the factors influencing migration of siloxanes, such as material composition, simulant polarity, and compound properties, and are supported by earlier reports on the thermal migration behavior of siloxanes [20,42].

### 3.4. Results of the Potential Exposure Assessment

#### 3.4.1. Total Migration Compliance Assessment

The total exposure results (Table 2) indicated a potential concern regarding the sum of cyclic siloxanes D3–D13, with several samples exceeding the proposed action limit of 12 mg/kg food for adults when tested with 95% ethanol (e.g., S1: 30.0 mg/kg, S33: 20.8 mg/kg) and all exceeding the 2 mg/kg limit for children, with the exception of sample 31. More critically, the total migration of siloxanes (D3–D13 and L3–L13) from four samples into 50% ethanol exceeded the overall limit of 60 mg/kg. Conversely, for the majority of samples, the total siloxane migration remained below this threshold, even when the sum of D3–D13 was exceeded, suggesting a lower contribution from linear siloxanes.

#### 3.4.2. Refined Risk Assessment of Individual Substances

In contrast, Table 3 provides complementary perspectives on the migration risk of siloxanes from silicone FCMs.

Table 3 presents a more granular risk assessment of the 18 detected migrant siloxanes, using RQ and TTC approaches for individual compounds across all age groups. Notably, RQs for D4 and D5 remained below 5 across all age groups, indicating negligible exposure risk. In contrast, RQ of D3 exceeded 5 in all individuals aged >13 years, primarily resulting from its relatively low RfD of 0.4 in combination with the substantial contribution of total food intake to the exposure estimate. Similarly, the higher RQ values observed for L4 in sample 1 across all age groups result from its even lower RfD (0.04). For the remaining 14 siloxanes, including L5, D6, and L6, the exposure levels all exceeded the TTC threshold of 0.0015 mg/kg bw/day across all age groups. The lowest value was observed for L8 in the 3–6-year-old group (S3: 0.20 mg/kg bw/day), while the highest was in the 1–3-year-old group (S22: 13.52 mg/kg bw/day).

The divergence between the two approaches underscores the importance of multi-dimensional risk assessment. Aggregate migration limits (Table 2) are useful for screening and compliance but may overlook compound-specific risk profiles. RQ and TTC analysis offers a more refined view of exposure risks, particularly for vulnerable age groups.

## 4. Conclusions

This study established a migration testing framework incorporating both routine and extreme exposure scenarios to investigate siloxane migration from silicone FCMs. Analysis of the 30 silicone FCMs detected migration of 27 of the 35 target siloxanes, with migration levels ranging from <LOD to 23.72 mg/kg. Exposure to the sum of cyclic siloxanes (D3–D13) in children exceeded the proposed action limit of 2 mg/kg in food for nearly all samples. D3 in several samples showed RQ > 5, especially for individuals aged >13 years. The TTC assessment of 14 siloxanes lacking reference doses demonstrated that all exceeded the threshold, with D6 and L12 being particularly prevalent (>90% detection) and of potential risk across all age groups.

Importantly, the migration data generated under the worst-case conditions used in this study represent a conservative upper bound of migration, which may be higher than the actual exposure levels of consumers under routine daily use conditions. That said, the conclusions of this study are limited to silicone FCMs sold on Chinese e-commerce platforms and cannot be directly extrapolated to products from other regions or offline retail channels. Future work should include offline retail channels and international products to enable cross-regional and cross-channel comparisons. In addition, further migration tests under real food matrices and daily use scenarios should be carried out to refine the actual consumer exposure assessment of siloxanes, bridging the gap between laboratory worst-case testing and real-world exposure scenarios. From a methodological perspective, the lack of commercial reference standards for some siloxanes may introduce uncertainty into the quantitative results. The risk assessment for unregistered compounds was based on the TTC approach, which may produce conservative risk estimates. Further toxicological studies are required to establish compound-specific toxicity thresholds and long-term exposure data for these siloxanes. Finally, regulatory frameworks for silicone FCMs should be iteratively updated based on emerging scientific evidence, with strengthened safety management to ensure adequate protection of consumer health.

## Figures and Tables

**Table 1:** Name, CAS number, molecular formula and weight, retention time and LOD of each targeted siloxane.

Acronym	Name	Molecular Formula	CAS Number	MW	Retention Time (min)	RI-Exp.	RI-Doc.	Qualitative–Quantitative Ion (*m*/*z*)	LOD (mg/kg)
Others									
MDHM ^a^	Heptamethyl trisiloxane	C_7_H_22_O_2_Si_3_	1873-88-7	221.50	6.03	841	NA	221, 73, 222	1.0
INCI ^a^	Drometrizole trisiloxane	C_24_H_39_N_3_O_3_Si_3_	155633-54-8	501.80	28.94	2927	NA	221, 73, 369	1.0
LMSs									
L2 ^a^	Hexamethyldisiloxane	C_6_H_18_OSi_2_	107-46-0	162.38	3.75	591	598	147,73,131	0.2
L3 ^a^	Octamethyltrisiloxane	C_8_H_24_O_2_Si_3_	107-51-7	236.53	6.63	878	NA	221,73, 205	0.1
L4 ^a^	Decamethyltetrasiloxane	C_10_H_30_O_3_Si_4_	141-62-8	310.68	9.66	1062	1062	207,73,295	0.1
L5 ^a^	Dodecamethylpentasiloxane	C_12_H_36_O_4_Si_5_	141-63-9	384.84	12.33	1235	1235	147, 73, 369	0.1
L6 ^a^	Tetradecamethylhexasiloxane	C_14_H_42_O_5_Si_6_	107-52-8	458.99	14.67	1402	1402	73, 221, 443	0.1
L7 ^a^	Hexadecamethylheptasiloxane	C_16_H_48_O_6_Si_7_	541-01-5	533.10	16.68	1565	1565	221, 73, 295	0.2
L8 ^a^	Octadecamethyloctasiloxane	C_18_H_54_O_7_Si_8_	556-69-4	607.30	18.54	1730	1730	73, 221, 295	0.2
L9 ^a,^*	Eicosamethylnonasiloxane	C_20_H_60_O_8_Si_9_	2652-13-3	681.50	20.38	1899	1890	221,147,73	0.5
L10 ^a^	Docosamethyldecasiloxane	C_22_H_66_O_9_Si_10_	556-70-7	755.60	20.20	2047	2047	73, 221, 147	1.0
L11 ^a,^*	Tetracosamethylundecasiloxane	C_24_H_72_O_10_Si_11_	107-53-9	829.80	23.32	2209	2200	221, 147, 73	1.0
L12 ^a,^*	Hexacosamethyldodecasiloxane	C_26_H_78_O_11_Si_12_	2471-08-1	903.90	24.52	2351	2350	221, 147, 73	1.0
L13 ^a,^*	Octacosamethyl tridecasiloxane	C_28_H_84_O_12_Si_13_	2471-09-2	978.10	25.72	2498	2497	221, 147, 73	1.0
L14 *	Triacontamethyltetradecasiloxane	C_30_H_90_O_13_Si_14_	2471-10-5	1052.20	26.82	2640	2643	221, 147, 73	1.0
CMSs									
D3 ^a^	Hexamethylcyclotrisiloxane	C_6_H_18_O_3_Si_3_	541-05-9	222.46	5.65	817	833	207, 96, 191	1.0
D4 ^a^	Octamethylcyclotetrasiloxane	C_8_H_24_O_4_Si_4_	556-67-2	296.61	8.54	990	990	281, 73, 191	0.1
D5 ^a^	Decamethylcyclopentasiloxane	C_10_H_30_O_5_Si_5_	541-02-6	370.77	11.03	1147	1147	355, 73, 267	0.1
D6 ^a^	Dodecamethylcyclohexasiloxane	C_12_H_36_O_6_Si_6_	540-97-6	444.92	13.56	1319	1319	73, 341, 429	0.1
D7 ^a^	Tetradecamethylcycloheptasiloxane	C_14_H_42_O_7_Si_7_	107-50-6	519.07	15.79	1492	1492	73, 281, 147	0.1
D8 ^a^	Hexadecamethylcyclooctasiloxane	C_16_H_48_O_8_Si_8_	556-68-3	593.20	17.85	1664	1664	355, 73, 221	0.1
D9 ^a^	Octadecamethylcyclononasiloxane	C_18_H_54_O_9_Si_9_	556-71-8	667.40	19.63	1826	1826	73, 355, 221	0.1
D10 ^a,^*	Eicosamethylcyclodecasiloxane	C_20_H_60_O_10_Si_10_	18772-36-6	741.50	21.16	1978	1981	281, 147, 207	0.5
D11 ^a,^*	Docosamethylcycloundecasiloxane	C_22_H_66_O_11_Si_11_	18766-38-6	815.70	22.59	2130	2134	355, 147, 281	0.5
D12 ^a,^*	Tetracosamethylcyclododecasiloxane	C_24_H_72_O_12_Si_12_	18919-94-3	889.80	23.91	2277	2281	355, 147, 429	0.5
D13 ^a,^*	Hexacosamethylcyclotridecasiloxane	C_26_H_78_O_13_Si_13_	23732-94-7	963.99	25.11	2423	2425	147, 73, 221	0.5
D14 *	Octacosamethylcyclotetradecasiloxane	C_28_H_84_O_14_Si_14_	149050-40-8	1038.10	26.24	2564	2568	147, 73, 355	0.5
D15 *	Triacontamethylcyclopentadecasiloxane	C_30_H_90_O_15_Si_15_	23523-14-0	1112.30	27.29	2701	2709	147, 73, 355	0.5
D16 *	Dotriacontamethylcyclohexadecasiloxane	C_32_H_96_O_16_Si_16_	150026-95-2	1186.50	28.53	2872	2857	147, 73, 221	0.5
D17 *	Tetratriacontamethylcycloheptadecasiloxane	C_34_H_102_O_17_Si_17_	150026-96-3	1260.60	29.34	2982	2984	147, 73, 221	0.5
D18 *	Hexatriacontamethylcyclooctadecasiloxane	C_36_H_108_O_18_Si_18_	23523-12-8	1334.77	30.32	3106	3115	147, 73, 221	1.0
D19 *	Octatriacontamethylcyclononadecasiloxane	C_38_H_114_O_19_Si_19_	150026-97-4	1408.90	31.51	3235	3247	281, 73, 147	1.0
D20 *	Tetracontamethylcycloeicosasiloxane	C_40_H_120_O_20_Si_20_	150026-98-5	1483.10	32.94	3365	3380	281, 73, 147	1.0
D21 *	Dotetracontamethylcycloheneicosasiloxane	C_42_H_126_O_21_Si_21_	23523-13-9	1557.20	34.69	3497	3512	281, 73, 147	1.0
D22 *	Tetratetracontamethylcyclodocosasiloxane	C_44_H_132_O_22_Si_22_	1177831-23-0	1631.40	37.14	3643	3642	281, 73, 147	1.0

Notes: : ^a^ for Compounds for which reference standards are available; * For siloxanes without reference standards, the identification is performed using the RI method; Xa: small-molecule siloxanes with a molecular weight < 1000 Da; Exp., experimental RI values in this study; Doc., RI values detected with the same stationary phase as the HP-5MS column from the literature and databases (the RI values for L4–L14 and D4–D22 are sourced from Di Feng et al. [5]; others are from the U.S. National Library of Medicine); NA: no relevant data found.

**Table 2:** Total exposure to low-molecular-weight siloxanes of D3–D13 and L3–L13.

Sample	Food Simulant	Sum of D3–D13, mg/kg Food		Sum of D3–D13 and L3–L13, mg/kg Food	
		Action Limit	Migration Level	Action Limit	Migration Level
No. 9	4% acetic acid	2	4.3	60	23.1
No. 31			<LOD		1.5
No. 3	50% ethanol	2	22.8		47.8
No. 4			24.2		61.9
No. 5			26.3		69.3
No. 6			13.3		27.0
No. 10			15.0		29.9
No. 14			4.9		12.1
No. 15			6.1		12.2
No. 16			10.2		21.4
No. 17			12.2		26.3
No. 19			13.5		26.9
No. 20			11.0		21.0
No. 21			13.1		26.1
No. 22			25.3		97.1
No. 24			10.6		21.7
No. 25			10.0		20.1
No. 26			12.3		24.7
No. 27			9.7		20.6
No. 28			10.4		24.0
No. 29			9.9		20.8
No. 30			9.0		19.1
No. 34			20.0		65.8
No. 1	95% ethanol	12 for adults, 2 for children	30.0		58.5
No. 2			15.0		30.5
No. 18		12	6.7		17.0
No. 32		12 for adults, 2 for children	20.5		45.3
No. 33			20.8		45.2
No. 35			19.0		39.4
No. 36			14.9		31.9

**Table 3:** Exposure evaluation of siloxanes (MW < 1000) migrated from 30 silicone FCMs into food simulants.

Sample	Age (Year)	Compounds																		
		D3	D4	L4	D5	TTC	L5	D6	L6	D7	L7	D8	L8	D9	D10	L10	L9	D11	L12	D13
		RQ					Exposure, mg/kg bw/day													
S9	<1				0.05	0.0015 mg/kg bw/day		0.45		0.29	6.05		1.16	1.02		0.60				
	1–3				0.06			0.54		0.35	7.36		1.41	1.24		0.73				
S31	<1										0.46		0.24							
	1–3										0.56		0.29							
S3	<1	9.96	0.44		0.45		0.78	1.01		0.24		0.24	0.23						1.31	
	1–3	12.12	0.53		0.55		0.96	1.24		0.29		0.30	0.29						1.61	
	3–6	8.31	0.37		0.38		0.66	0.85		0.20		0.20	0.20						1.11	
S4	<1	8.64	0.50		0.28		1.36	1.21			1.39	0.30	3.09		1.03	0.93	0.61		2.05	
	1–3	10.51	0.61		0.34		1.66	1.47			1.69	0.37	3.76		1.25	1.13	0.75		2.49	
	3–6	7.21	0.42		0.24		1.14	1.01			1.16	0.25	2.58		0.86	0.78	0.51		1.71	
S19	<1	8.61	0.16		0.06			0.27											1.68	
	1–3	4.91	0.09		0.04			0.33											2.05	
	3–6	3.37	0.06		0.02			0.22											1.40	
S21	<1	7.35	0.28					0.69											1.45	
	1–3	4.19	0.16					0.84											1.76	
	3–6	2.88	0.11					0.58											1.21	
S34	<1	5.81	0.50		0.37		0.78	1.24	0.49	0.69	1.08	0.30	6.63	0.33		3.49			0.75	
	1–3	7.07	0.61		0.45		0.95	1.51	0.60	0.84	1.32	0.37	8.06	0.40		4.25			0.91	
	3–6	4.85	0.42		0.31		0.65	1.03	0.41	0.58	0.90	0.25	5.53	0.28		2.91			0.63	
S5	<1	10.40	0.49		0.34		1.65	1.94		0.48	0.67	0.37	5.38		1.37	1.46			0.53	
	1–3	2.77	0.24		0.18		2.01	2.36		0.58	0.82	0.45	6.55		1.66	1.78			0.65	
S6	<1	7.64	0.25		0.09		0.23	0.64				0.30							0.88	
	1–3	9.29	0.30		0.11		0.29	0.78				0.36							1.07	
S10	<1	11.05	0.23					0.51		0.44		0.35							0.45	
	1–3	13.44	0.28					0.62		0.54		0.42							0.55	
S14	<1	2.61	0.15					0.30			0.28		0.54			0.28			0.39	
	1–3	3.18	0.18					0.36			0.34		0.66			0.34			0.47	
S15	<1	1.95	0.11					0.60											0.68	
	1–3	2.37	0.13					0.73											0.83	
S20	<1	7.82	0.17					0.35						0.27	0.23				0.56	
	1–3	9.51	0.21					0.42						0.33	0.28				0.68	
S22	<1	7.95	0.48		0.35		0.73	1.67	0.73	0.49	4.66	0.83	11.12		1.51	5.03	1.00		0.67	
	1–3	9.66	0.58		0.43		0.89	2.03	0.89	0.59	5.66	1.01	13.52		1.84	6.12	1.21		0.82	
S24	<1	5.38	0.14		0.15			0.44			0.23	0.38							0.73	
	1–3	6.54	0.17		0.18			0.53			0.29	0.46							0.89	
S25	<1	5.17	0.25					0.32											1.40	
	1–3	6.29	0.30					0.39											1.70	
S26	<1	7.18	0.14					0.48		0.47		0.25							1.19	
	1–3	8.74	0.17					0.59		0.58		0.30							1.45	
S27	<1	5.53	0.21					0.28			0.26		0.36						1.28	
	1–3	6.73	0.25					0.34			0.31		0.43						1.55	
S28	<1	1.96	0.19		0.18		0.40	0.78				0.24	0.74			0.33			1.50	
	1–3	2.38	0.23		0.22		0.49	0.95				0.30	0.90			0.40			1.82	
S29	<1	2.13	0.14		0.05		0.46	0.62				0.26							2.11	
	1–3	2.59	0.17		0.06		0.56	0.76				0.31							2.57	
S30	<1	2.27	0.23		0.17		0.52	0.86											0.75	
	1–3	2.77	0.28		0.21		0.63	1.04											0.92	
S16	1–3	2.74	0.15		0.21			1.56		0.39	0.60	0.36							0.78	
	3–6	1.88	0.10		0.15			1.07		0.27	0.41	0.25							0.54	
	6–13	4.33	0.24		0.34			2.46		0.62	0.96	0.57							1.24	
	13–20	6.62	0.36		0.52			3.77		0.95	1.46	0.87							1.89	
	20–50	8.23	0.45		0.64			4.68		1.18	1.82	1.08							2.35	
	>50	7.47	0.41		0.58			4.25		1.07	1.65	0.98							2.13	
S17	<1	2.30	0.10		0.17		0.94	1.75		0.44	0.25	0.47		0.25					0.63	
	1–3	2.79	0.12		0.21		1.15	2.13		0.54	0.30	0.58		0.30					0.76	
	3–6	1.92	0.08		0.14		0.79	1.46		0.37	0.21	0.40		0.21					0.52	
	6–13	4.42	0.19		0.33		1.81	3.37		0.85	0.48	0.91		0.48					1.21	
	13–20	6.76	0.29		0.51		2.77	5.16		1.30	0.73	1.39		0.73					1.85	
	20–50	8.41	0.37		0.63		3.45	6.42		1.61	0.91	1.73		0.91					2.30	
	>50	7.63	0.33		0.57		3.13	5.82		1.46	0.83	1.57		0.83					2.09	
S1	1–3	4.22	0.25	13.26	0.63			4.28		2.17		0.77	0.31	1.27		0.46		0.86	1.98	
	3–6	2.89	0.17	9.09	0.43			2.94		1.49		0.53	0.21	0.87		0.31		0.59	1.36	
	6–13	6.68	0.40	20.98	0.99			6.78		3.43		1.22	0.49	2.00		0.72		1.35	3.13	
	13–20	10.21	0.61	32.08	1.52			10.36		5.24		1.86	0.75	3.06		1.10		2.07	4.79	
	20–50	12.70	0.76	39.89	1.89			12.88		6.52		2.32	0.93	3.81		1.37		2.57	5.95	
	>50	11.52	0.69	36.20	1.72			11.69		5.92		2.10	0.84	3.46		1.25		2.34	5.40	
S2	1–3	2.61	0.61		0.35			1.11		0.30	0.41		0.38	0.49					1.60	
	3–6	1.79	0.42		0.24			0.76		0.21	0.28		0.26	0.34					1.10	
	6–13	4.13	0.96		0.56			1.75		0.48	0.65		0.60	0.78					2.53	
	13–20	6.31	1.47		0.85			2.68		0.73	0.99		0.92	1.19					3.86	
	20–50	7.85	1.83		1.06			3.33		0.91	1.24		1.15	1.48					4.80	
	>50	7.12	1.66		0.96			3.02		0.83	1.12		1.04	1.34					4.36	
S18	13–20	14.39	0.72					0.88	0.98		2.15		1.77							
	20–50	17.89	0.89					1.10	1.22		2.68		2.20							
	>50	16.23	0.81					1.00	1.11		2.43		1.99							
S32	1–3	5.79	0.51		0.22		0.35	0.98	0.31	0.56	0.30	0.46	0.92	0.32		2.14		1.24	2.80	
	3–6	3.97	0.35		0.15		0.24	0.67	0.21	0.39	0.21	0.32	0.63	0.22		1.47		0.85	1.92	
	6–13	9.16	0.80		0.35		0.56	1.55	0.49	0.89	0.48	0.73	1.46	0.51		3.38		1.96	4.43	
	13–20	14.01	1.23		0.54		0.86	2.37	0.75	1.37	0.73	1.12	2.24	0.77		5.18		2.99	6.78	
	20–50	17.41	1.53		0.67		1.06	2.95	0.93	1.70	0.91	1.39	2.78	0.96		6.43		3.72	8.42	
	>50	15.81	1.39		0.61		0.97	2.68	0.84	1.54	0.83	1.26	2.52	0.87		5.84		3.38	7.65	
S33	1–3	3.35	0.22		0.81		0.93	0.65	0.43	1.02	0.42	0.40	0.67	0.42		1.05		1.01	2.13	
	3–6	2.30	0.15		0.56		0.64	0.45	0.29	0.70	0.29	0.28	0.46	0.29		0.72		0.69	1.46	
	6–13	5.30	0.35		1.28		1.47	1.03	0.68	1.62	0.67	0.64	1.06	0.67		1.66		1.60	3.37	
	13–20	8.11	0.54		1.96		2.25	1.57	1.04	2.47	1.02	0.98	1.61	1.02		2.54		2.44	5.16	
	20–50	10.08	0.67		2.44		2.80	1.96	1.29	3.07	1.27	1.22	2.01	1.27		3.16		3.04	6.42	
	>50	9.15	0.61		2.21		2.54	1.78	1.17	2.79	1.15	1.11	1.82	1.15		2.87		2.76	5.82	
S35	1–3	4.22	0.37		0.37			0.96		1.13	0.54	0.42	0.73	0.47		0.72		0.76	2.24	
	3–6	2.89	0.25		0.25			0.66		0.77	0.37	0.29	0.50	0.32		0.50		0.52	1.54	
	6–13	6.68	0.58		0.58			1.53		1.79	0.85	0.66	1.16	0.74		1.15		1.21	3.55	
	13–20	10.21	0.88		0.89			2.33		2.73	1.30	1.01	1.77	1.13		1.75		1.85	5.42	
	20–50	12.70	1.10		1.10			2.90		3.40	1.61	1.25	2.20	1.41		2.18		2.30	6.74	
	>50	11.52	1.00		1.00			2.63		3.08	1.46	1.14	1.99	1.28		1.98		2.09	6.12	
S36	1–3	2.48	0.18		0.24		0.42	0.42		0.82	0.34	0.53	0.90	0.73		0.43	1.68	0.89	1.31	0.92
	3–6	1.70	0.12		0.19		0.29	0.29		0.56	0.23	0.36	0.62	0.50		0.29	1.15	0.61	0.90	0.63
	6–13	3.93	0.28		0.39		0.66	0.66		1.29	0.53	0.84	1.43	1.16		0.68	2.65	1.41	2.08	1.45
	13–20	6.00	0.43		0.59		1.01	1.01		1.97	0.81	1.28	2.18	1.77		1.04	4.06	2.15	3.17	2.22
	20–50	7.46	0.53		0.74		1.25	1.25		2.45	1.01	1.60	2.71	2.20		1.29	5.04	2.68	3.95	2.76
	>50	6.77	0.48		0.67		1.14	1.14		2.23	0.92	1.45	2.46	1.99		1.17	4.58	2.43	3.58	2.51

Notes: RfD of D3, D4, L4 and D5 is 0.4, 3.7, 0.04, and 5, respectively. Individual’s total food intake from [45] , EPA, g food/kg bw/day.

## Data Availability

The original contributions presented in this study are included in the article/Supplementary Materials. Further inquiries can be directed to the corresponding author.

## Supplementary Materials

The following supporting information can be downloaded at: https://www.mdpi.com/article/10.3390/foods15081387/s1, Table S1: Sample information and conditions used for migration; Table S2: Contents of 24 siloxanes detected in 30 FCMs extracted by acetone (n = 3); Table S3: Migration levels of the 27 siloxanes from 30 silicone FCMs into food simulants (mg/kg food) (n = 3). Figure S1: Classification diagram of silica gel samples. A is the distribution chart of silica gel sample categories, and B is the classification chart of silica gel sample sources.

## Abbreviations

The following abbreviations are used in this manuscript:

BW/bw	Body Weight
CF	Consumption Factor
CMSs	Cyclic Methylsiloxanes
DFs	Detection Frequencies
ECHA	European Chemicals Agency
EI	Electron Ionization
EPA	U.S. Environmental Protection Agency
EU	The European Union
FCMs	Food Contact Materials
FDA	U.S. Food and Drug Administration
GC-MS	Gas Chromatography—Mass Spectrometry
LMSs	Linear Methylsiloxanes
LOD	Limit of Detection
LOQ	Limit of Quantification
MW	Molecular Weight
NA	Not Applicable (No Relevant Data Found)
PCA	Principal Component Analysis
PBT	Persistent, Bioaccumulative, and Toxic
POPs	Persistent Organic Pollutants
PTFE	Polytetrafluoroethylene
REACH	Registration, Evaluation, Authorization and Restriction of Chemicals
RfD	Reference Dose
RI	Retention Index
RQ	Risk Quotient
RSD	Relative Standard Deviation
SIM	Selected Ion Monitoring
SVHCs	Substances of Very High Concern
TICs	Total Ion Chromatograms
TTC	Threshold of Toxicological Concern
vPvB	very Persistent very Bioaccumulative
