# Supplementary material for: Migration of 35 Siloxanes from Silicone Food Contact Materials in China and Their Potential Exposure Assessment
Source: Foods. 2026 Apr 16;15(8):1387. doi: 10.3390/foods15081387 (PMC13115124; doi:10.3390/foods15081387)
Supplement: Supplementary file 1 [file foods-15-01387-s001.zip › foods-4211877-supplementary.pdf]

Supplementary Materials

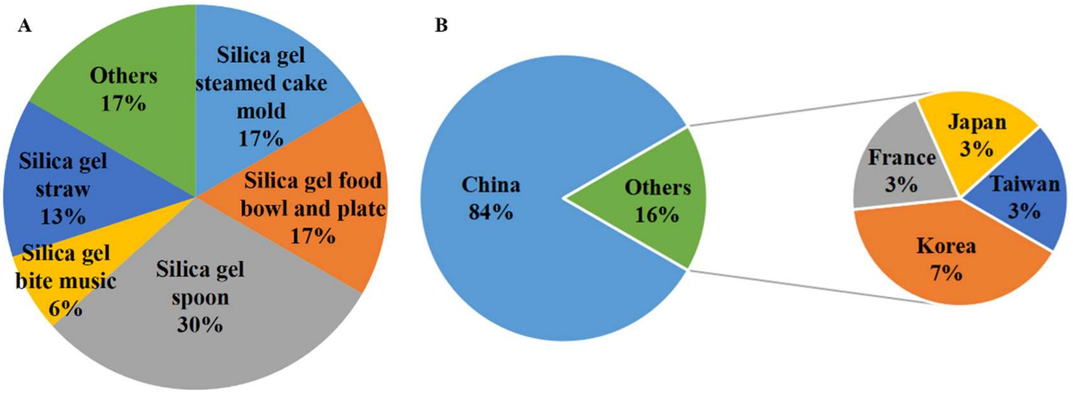

Figure S1 Classification diagram of silica gel samples. A is the distribution chart of silica gel sample categories, and B is the classification chart of silica gel sample sources.

Table S1 Sample information and conditions used for migration

No.	FCMs Sample name	Sort	Food simulant				Age group	Conditions used for migration test
			4% acetic acid	10% ethanol	50% ethanol	95% ethanol		
S1	Steamed cake mould	Mold			√	√	≥1 year	95% ethanol at 70 °C for 2 h
S32	Steamed cake mould				√	√		
S33	Steamed cake mould				√	√		
S35	Steamed cake mould				√	√		
S36	Steamed cake mould				√	√		
S3	silica gel food bowl	Bowl and Plate	√	√	√		3 months ~6 years	50% ethanol at 70 °C for 2 h
S4	silica gel food plate		√	√	√			
S19	silica gel food plate		√	√	√			
S21	silica gel food bowl		√	√	√			
S34	silica gel food plate		√	√	√			
S5	silica gel spoon	Spoon	√	√	√		3 months ~3 years	50% ethanol at 70 °C for 2 h
S6	Temperature-sensing spoon		√	√	√			
S10	Baby rice batter spoon		√	√	√			
S20	silica gel soft spoon		√	√	√			
S22	Complementary food spoon		√	√	√			
S24	Soft baby spoon		√	√	√			
S25	silica gel spoon		√	√	√			
S26	silica gel spoon		√	√	√			
S27	silica gel spoon		√	√	√			
S9	Complementary food bite bag	Bite bag	√				3 months ~3 years	4% acetic acid at 40 °C for 2 h
S31	food bite bag		√					
S28	Soup straw	Straw	√	√	√		6 months ~ 3 years	50% ethanol at 70 °C for 2h
S29	silica gel straw for kids		√	√	√			
S30	silica gel straw		√	√	√			

S15	silica gel straw		√	√	√			
S2	silica gel spatula	Others	√	√	√	√	≥1 year	50% ethanol for S14, S16, S17, 95% ethanol for S2 and S18, at 70 ℃ for 2h
S14	Baby bottle pacifier			√	√		1 month ~ 3 years	
S16	silica gel cup			√	√		≥1 year	
S17	Thermos cup seal ring			√	√		≥1 month	
S18	Rubber gloves		√	√	√	√	≥13 years	

Table S2 Contents of 24 siloxanes detected in 30 FCMs extracted by acetone (n=3)

Sample	Compounds, mg/kg																							
	D3	D4	D5	D6	D7	D8	D9	L9	L10	D10	D11	D12	L12	D13	L13	D14	L14	D15	D16	D17	D18	D19	D20	D21
S1	66.2	615.6	806.8	760.5	377.5	245.2	238.7	ND	ND	1480	1513	1787	ND	1957	ND	2342	ND	2548	2530	2362	1964	1518	1118	815.5
S2	155.4	598.6	826.4	793.7	410.5	270.6	262.2	ND	ND	1516	1710	1647	ND	1748	ND	1992	ND	2158	2114	1896	1670	1199	856.4	601.2
S3	113.5	778.2	1086	923.5	380.6	215.7	206.5	ND	ND	1207	1225	1391	ND	1535	ND	1846	ND	1987	1915	1719	1493	1090	832.2	597.3
S4	334.9	770.5	1004	845.7	374.6	234.4	225.9	ND	101.7	1325	1309	1498	ND	1632	ND	1905	ND	2088	2044	1812	1508	1205	841.1	669.9
S5	105.5	294.1	643.7	843.8	551.2	390.3	349.7	91.9	ND	1761	1693	1683	ND	1767	ND	2140	ND	2085	2051	1842	1550	1097	806.1	614.7
S6	10.0	3.4	11.6	3.2	ND	ND	ND	ND	865.1	145.3	ND	ND	ND	ND	ND	ND	ND	ND	ND	ND	ND	ND	ND	ND
S9	12.4	8.2	13.3	18	18.4	25.4	40.3	25.6	25.1	318.9	494.7	726.4	50.3	1020	ND	1448	ND	1655	1751	1561	1383	1050	862.1	583.4
S10	6.6	ND	ND	ND	ND	ND	ND	ND	ND	ND	15.6	40.7	ND	107.0	ND	260.1	ND	467.5	746.7	879.1	948.2	840.0	683.6	558.4
S14	9.9	ND	11.3	11.6	24.6	40.1	57.0	ND	ND	401.5	484.1	660.6	ND	917.2	ND	1299	ND	1627	1832	1691	1558	1213	907.1	693.2
S15	19.9	32.8	177	265	189.8	150.9	162.3	ND	ND	1138	1087	1235	ND	1347	ND	1588	ND	1740	1668	1483	1277	949.1	745.8	553.5
S16	55.2	91.6	294.7	430.1	270.6	187.7	183.6	ND	ND	1201	1129	1250	ND	1312	ND	1312	ND	1636	1578	1381	1128	824.7	574.5	450.7
S17	36.2	78.5	425.7	626.7	378.7	255.8	247.4	ND	ND	1676	1544	1728	ND	1747	ND	2083	ND	2251	2189	1935	1643	1169	850.8	629.7
S18	3.7	ND	4.0	2.2	ND	ND	ND	ND	347.9	ND	326.4	508.6	ND	ND	5717	ND	ND	ND	ND	ND	ND	ND	ND	ND
S19	10.9	ND	33.7	62.1	47.8	46.2	62.4	ND	ND	442.4	573.0	787.5	ND	994.0	ND	1348	ND	1672	1750	1683	1463	1217	860.1	692.1
S20	ND	ND	ND	ND	ND	ND	ND	ND	ND	ND	ND	ND	ND	ND	ND	ND	ND	ND	ND	ND	ND	145.0	164.3	107.4
S21	6.7	ND	233.6	582.7	397.8	289.5	280.6	ND	ND	1561	1472	1614	ND	1665	ND	1965	ND	2148	2066	1938	1555	1150	859.2	713.7
S22	72.2	459.1	926.9	863.9	425.0	274.3	258.1	ND	1075	1426	1382	1488	440.0	1498	262.3	1703	147.7	1880	1811	1638	1405	1081	774.4	564.7
S24	ND	ND	ND	6.0	15.1	22.8	48.9	ND	21.7	437.8	518.1	681.5	ND	853.4	21.6	1185	ND	1548	1719	1771	1627	1357	1066	815.7
S25	10.0	ND	119.8	261.5	154.8	82.2	60.1	ND	ND	280.0	272.4	323.3	ND	406.6	ND	577.6	ND	771.9	949.9	967.7	975.0	873.1	711.8	544.4
S26	4.7	ND	38.0	75.2	49.9	32.4	32.9	ND	ND	231.8	305.0	482.6	ND	730.5	ND	1062	ND	1308	1436	1335	1272	959.1	706.7	560.7
S27	4.9	ND	21.2	27.2	22.3	17.3	19.5	ND	ND	132.0	196.2	352.8	ND	599.5	ND	975.0	ND	1323	1504	1520	1350	1108	716.3	623.1
S28	92.9	331.3	499.3	470.4	281.7	200.1	201.4	ND	48.4	1201	1162	1318	ND	1383	ND	1591	ND	1751	1692	1522	1323	986.4	751.5	550.5

S29	13.8	ND	118.9	278.9	236	199.2	214.1	ND	ND	1237	1254	1406	ND	1405	ND	1839	ND	1973	1972	1763	1526	1177	838.0	662.7
S30	85.3	348.5	638.4	664.5	350.5	212.9	192.9	ND	ND	1148	1089	1246	ND	1204	ND	1583	ND	1718	1673	1484	1267	946.9	747.1	474.9
S31	ND	ND	ND	ND	ND	ND	ND	ND	ND	ND	ND	10.0	ND	59.3	ND	144.4	ND	274.3	404.1	415.4	431.7	276.3	239.6	188.8
S32	20.8	19.6	31.9	65.8	66.6	61.5	79.2	ND	19.1	676.2	991.2	725.2	ND	938.8	ND	975.8	ND	904.2	805.2	632.8	521.3	349.3	247.1	210.4
S33	1.9	35.8	93.8	162.2	161.9	129.0	133.9	ND	ND	983.2	1061	1170	ND	1390	ND	1530	ND	1460	1304	1075	826.0	571.6	385.4	311.5
S34	88.4	187.9	273.1	195	107.6	66.7	69.0	ND	149.2	507.0	488.9	527.6	ND	605.9	ND	640.4	ND	587.1	530.9	406.1	327.7	247.2	191.8	133.6
S35	30.7	179.2	373.6	367	231	137.7	115.5	ND	45.4	765.4	674.7	692.7	ND	798.0	ND	853.1	ND	823.1	753.2	6083	499.3	348.3	249.2	156.1
S36	46.7	207.6	388.0	338.5	214	133.8	117.2	ND	ND	823.9	1021	758.8	ND	888.5	ND	974.7	ND	949.2	848.7	693.9	580.5	409.7	279.4	229.2

Sum of cyclic siloxanes and linear siloxanes, mg/kg

Sample	$\sum CMS$	$\sum LMS$	Sample	$\sum CMS$	$\sum LMS$	Sample	$\sum CMS$	$\sum LMS$	Sample	$\sum CMS$	$\sum LMS$
S1	25046		S14	13439		S22	19929	1925	S31	2444	
S2	22426		S15	15809		S24	13673	43	S32	8323	19
S3	20541		S16	15290		S25	8342		S33	12788	
S4	21629	102	S17	21496		S26	10623		S34	6182	149
S5	22268	92	S18	845	6065	S27	10512		S35	14130	45
S6	174	865	S19	13746		S28	17309	48	S36	9904	
S9	12988	101	S20	417		S29	18116				
S10	5554		S21	20498		S30	17074				

Note: ND means <LOD

Table S3 Migration levels of the 27 siloxanes from 30 silicone FCMs into food simulants (mg/kg food)(n=3)

Simulant	Sample	Compounds																										
		D3	D4	L4	D5	L5	D6	L6	D7	L7	D8	L8	D9	D10	L10	L9	D11	L12	D13	D14	L14	D15	D17	D18	D19	D20	D21	D22
4% acetic acid	No.9				0.56		0.95		0.61	12.9		2.47	2.18		1.28													
	No.31									0.99		0.51																
50% ethanol	No.3	8.50	3.46		4.80	1.69	2.17		0.51		0.52	0.50						2.83										
	No.4	7.37	3.97		3.02	2.91	2.58			2.97	0.65	6.60		2.20	1.99	1.31		4.37										
	No.5	8.87	3.83		3.63	3.52	4.14		1.02	1.43	0.79	11.48		2.92	3.12			1.14										
	No.6	6.52	1.95		0.93	0.50	1.37				0.63							1.87						0.94				
	No.10	9.43	1.81				1.08		0.94		0.74							0.97										
	No.14	2.23	1.19				0.63			0.60		1.15			0.59			0.83										
	No.15	1.66	0.83		0.89		1.28											1.46										
	No.16	1.92	0.97		1.88		2.73		0.69	1.06	0.63							1.37										
	No.17	1.96	0.79		1.84	2.01	3.74		0.94	0.53	1.01		0.53					1.34										
	No.19	7.35	1.29		0.66		0.57											3.59							1.59			
	No.20	6.67	1.35				0.74						0.57	0.49				1.19							0.65			
	No.21	6.28	2.21				1.47											3.09							1.18	1.08		
	No.22	6.78	3.75		3.77	1.56	3.56	1.56	1.04	9.93	1.77	23.72		3.22	10.73	2.13		1.43			1.27			0.66	1.57			
	No.24	4.59	1.13		1.58		0.93			0.50	0.81							1.56										
	No.25	4.41	1.96				0.68											2.98								0.93		
	No.26	6.13	1.09				1.03		1.01		0.53							2.54								0.90	1.40	0.54
	No.27	4.72	1.63				0.59			0.55		0.76						2.72										0.99
	No.28	1.67	1.48		1.91	0.86	1.66				0.52	1.58			0.71			3.20							2.63			
	No.29	1.82	1.12		0.57	0.98	1.33				0.55							4.51										
	No.30	1.94	1.79		1.84	1.11	1.83											1.61							2.41	5.03		
	No.34	4.96	3.96		3.96	1.67	2.64	1.05	1.47	2.31	0.65	14.14	0.71		7.45			1.60										

95% ethanol	No.1	2.96	1.64	0.93	5.51		7.51		3.80		1.35	0.54	2.22		0.80		1.50	3.47		2.53		2.55	2.52	1.62			3.68	
	No.2	1.83	3.94		3.08		1.94		0.53	0.72		0.67	0.86					2.80							1.70			
	No.18	4.17	1.92				0.64	0.71		1.56		1.28													1.38			
	No.32	4.06	3.30		1.95	0.62	1.72	0.54	0.99	0.53	0.81	1.62	0.56		3.75		2.17	4.91		2.57		2.54	2.57	1.53			3.49	
	No.33	2.35	1.45		7.11	1.63	1.14	0.75	1.79	0.74	0.71	1.17	0.74		1.84		1.77	3.74		1.92		1.86	1.97	1.19			3.74	
	No.35	2.96	2.37		3.22		1.69		1.98	0.94	0.73	1.28	0.82		1.27		1.34	3.93		1.98		1.96	2.02	1.27			3.78	
	No.36	1.74	1.14		2.15	0.73	0.73		1.43	0.59	0.93	1.58	1.28		0.75	2.94	1.56	2.30	1.61	1.79		1.77	1.85	1.17	2.40		2.86	
DF%		93.3	93.3	3.3	70.0	43.3	96.7	16.7	50.0	53.3	63.3	66.7	36.7	13.3	43.3	10.0	16.7	90.0	3.3	16.7	3.3	16.7	16.7	23.3	36.7	13.3	20.0	6.7

.
